# Cross-disease biomarker identification reveals shared diagnostic biomarkers for IVDD and NAFLD via bulk and single-cell RNA sequencing

**DOI:** 10.3389/fnmol.2025.1639705

**Published:** 2025-09-22

**Authors:** Jiasen Wei, Chenglong Ji, Lina Liu, Chen Yan, Linhui Han, Wenbo Lin, Ximing Xu, Kaiqiang Sun

**Affiliations:** ^1^Department of Spinal Surgery, Xinxiang Central Hospital, Xinxiang, China; ^2^The Fourth Clinical College of Xinxiang Medical University, Xinxiang, China; ^3^Department of Orthopedic Surgery, Changzheng Hospital, Navy Medical University, Shanghai, China; ^4^Department of Orthopedics, Naval Medical Center of PLA, Shanghai, China

**Keywords:** intervertebral disk degeneration, non-alcoholic fatty liver disease, machine learning, hub genes, inflammation, exercise

## Abstract

**Introduction:**

Intervertebral disc degeneration (IVDD) and non-alcoholic fatty liver disease (NAFLD) represent major global health burdens. Although recent evidence points to a potential association between these two conditions, the underlying molecular mechanisms remain poorly understood. This study aims to elucidate their shared molecular landscape using integrated bioinformatics approaches.

**Methods:**

Three IVDD and two NAFLD datasets were acquired from the Gene Expression Omnibus (GEO). We performed differential expression analysis (DEGs), weighted gene co-expression network analysis (WGCNA), and machine learning to identify shared hub genes. The diagnostic relevance of these genes was further assessed using ROC curves and nomograms. Single-cell sequencing analysis was employed to examine gene expression patterns across cell clusters in intervertebral disk and liver tissues. *In vivo* experiments were conducted to evaluate the influence of NAFLD on IVDD progression and the therapeutic potential of exercise intervention.

**Results:**

Six shared genes were identified between IVDD and NAFLD. Among these, ME1, HAS2, and ADRB2 were highlighted as potential biomarkers. Validation confirmed consistent expression patterns and strong predictive performance for both diseases. KEGG pathway and immune infiltration analyses indicated significant involvement of these biomarkers in disease-related pathways and immune cell interactions. Single-cell sequencing revealed distinct expression profiles and functional roles of ME1, HAS2, and ADRB2 across relevant cell types. *In vivo* studies demonstrated that NAFLD exacerbates IVDD progression, and intervention through swimming exercise ameliorated NAFLD and exerted protective effects on IVDD under high-fat diet conditions.

**Discussion:**

This study identifies ME1, HAS2, and ADRB2 as pivotal shared biomarkers for IVDD and NAFLD, providing new insights into their molecular interconnection. The findings enhance our understanding of the comorbid mechanisms and highlight the potential of exercise as a therapeutic strategy for both conditions. These results pave the way for further mechanistic and clinical research into common pathways and integrated treatment approaches.

## Introduction

Intervertebral disk degeneration (IVDD) is a chronic and degenerated diseases in spine field and has been proved to be the major contributor to low back pain (LBP), which imposes numerous society and economic burden worldwide (Risbud and Shapiro, 2014). In current clinical practice, although IVDD can be treated through conservative or surgical therapy, IVDD cannot be completely cured (Dowdell et al., 2017). Conservative therapy, such as physical therapy and oral medication, is mainly used in the early stage of IVDD and symptoms relief (Dowdell et al., 2017; Roh et al., 2021). Surgical treatment will be recommended for advanced IVDD, but with various complications, such as cerebrospinal fluid leakage and adjacent segment degeneration (Dowdell et al., 2017). Therefore, elucidating the diagnostic signatures and molecular mechanism for IVDD is clinically meaningful.

Non-alcoholic fatty liver disease (NAFLD) is also a prevalent chronic liver disease, with an incidence rate of 25 to 30% worldwide (Younossi, 2019). It is characterized by an abnormal deposition of triglycerides in liver (Lian et al., 2020). Advanced NAFLD can developed into liver cirrhosis and even hepatocellular tumors, imposing an escalating strain on healthcare systems (Cotter and Rinella, 2020). Various factors can result in NAFLD, such as lifestyle choices, dietary patterns, and genetic tendencies (Cotter and Rinella, 2020). The rising global occurrence of these disorders has highlighted their impact on liver health and their connection to extrahepatic issues, notably metabolic and neurological disturbances.

Recent researches have suggested a potential correlation between IVDD and NFALD (Zhou et al., 2024). Pathologically, IVDD is a progressive inflammation-related disease, where immune cell infiltration, particularly macrophages, plays a pivotal role in initiating a cascade of local inflammation and cell death (Koroth et al., 2023). Similarly, NAFLD is also a chronic liver disease and inflammatory response is also the major factor for dysfunction of hepatocytes (Younossi et al., 2018). In fact, due to the improvement of living standards, especially the substantial intake of high-fat diets, NAFLD is frequently linked with a spectrum of metabolic disorders, including obesity, hypertension, and diabetes, which in turn are also risk factors for IVDD (Francisco et al., 2022). Previous study indicated that LBP in patients with liver disease is a new-booming issue that required clinical attention (Bednár et al., 2023). In addition, a recent study reported that NAFLD was a risk factor for IVDD (Fu et al., 2024).

Despite a growing body of evidence having suggested a close link between IVDD and NAFLD, the precise molecular mechanisms remain obscure. Moreover, there is a significant gap in the comprehensive understanding of the shared diagnostic indicators between the two diseases. Therefore, this study aimed to employ bioinformatic methodologies to comprehensively screen the potential biomarkers associated with these two conditions. In addition, while our primary aim was to uncover shared mechanisms, we also evaluated the diagnostic potential of these genes through disease-specific nomograms, offering actionable insights for clinicians managing NAFLD or IVDD independently. The findings of this study would serve as a foundational theory to enhance our knowledge of diagnostic methods and treatment approaches for these intricate and interconnected health issues.

## Materials and methods

### Data collection

Totally five datasets related to IVDD (GSE153761, GSE56081, and GSE70362) and NAFLD (GSE63067 and GSE89632) were downloaded from the Gene Expression Omnibus (GEO) database.^1^ The selection criteria for the datasets were established as follows: (1) they must concentrate on either IVDD or NAFLD; (2) include both control and disease groups; and (3) provide access to raw data from GEO. Furthermore, we gathered additional signal cell sequencing data from GEO specifically for IVDD (GSE244889) and NAFLD (GSE202379). The entire analytical process is illustrated in Supplementary Figure S1.

### Differential gene expression (DEGs) analysis

We use the “limma” R package to visualize DEGs for IVDD and NAFLD groups. A significance level was established at |log2 FC| ≥ 0.5 and *p*-adjust < 0.05.

### Weighted gene co-expression network analysis (WGCNA)

We established gene co-expression networks and identified functional modules with the help of “WGCNA” R package (Langfelder and Horvath, 2008). Briefly, after eliminating outliers, we constructed a correlation matrix. Then, A topological overlap matrix (TOM) was then constructed by transforming the correlation matrix into an adjacency matrix. The TOM-based phase dissimilarity metric facilitated the clustering of genes with similar expression profiles into gene modules using average linkage hierarchical clustering. Lastly, we extracted relevant gene information from the corresponding modules to assess the relationship between gene significance (GS) and module membership (MM).

### Identification of the shared genes

The crucial shared genes for IVDD and NAFLD were identified by intersecting DEGs and selected module genes generated from WGCNA.

### Machine learning to identify the hub genes for IVDD and NAFLD

To pinpoint the crucial genes shared between IVDD and NAFLD, we subsequently deployed two machine learning models: the least absolute shrinkage and selection operator (LASSO) and the random forest (RF). LASSO regression was employed to (a) account for potential collinearity among the six candidate genes, and (b) cross-validate RF-derived gene rankings. Although LASSO is typically used for high-dimensional data, we used LASSO not as a standalone tool but in conjunction with RF in this study. Similar approaches have been validated in studies with small gene panels (Jiang et al., 2024; Lu et al., 2025; Chen et al., 2024; Han et al., 2024).

The LASSO algorithm was implemented using the R package “glmnet” (Friedman et al., 2010), and RF algorithm was carried out via the R package “randomForest” (Abegaz et al., 2023). Subsequently, a Venn diagram revealed four common genes for the two diseases. Through the analysis of these common genes, we identified three potential diagnostic targets. We also constructed receiver operating characteristic (ROC) curves through the “pROC” R package and visualized them with the “ggplot2” package to assess the diagnostic accuracy of the three hub genes (Jiang et al., 2024).

### Nomogram

We developed a nomogram model to predict IVDD and NAFLD progression based on key genes (Jiang et al., 2024). Subsequently, ROC analysis was performed to evaluate the performance of both the genetic signature and the nomogram models (Jiang et al., 2024). Calibration curves were used to assess the predictive accuracy. The “ggDCA” R package was used to generate decision curve analysis (DCA) curves for both the genetic signature and nomogram models (Jiang et al., 2024).

### Immune infiltration analysis

We utilized the single-sample gene set enrichment analysis (ssGSEA) algorithm to quantify the prevalence of 28 distinct immune cell populations via the GSVA package and its ‘gsva’ function. Furthermore, we calculated the Pearson correlation coefficient between the key hub genes and immune cells.

### Functional-enrichment analysis

For Kyoto Encyclopedia of Genes and Genomes (KEGG) pathway annotations, we utilized the KOBAS-i online tool, which can be accessed at http://bioinfo.org/kobas/. A false discovery rate (FDR) of less than 0.05 was considered statistically significant (Chai et al., 2020).

### Establishment of the NAFLD mouse model with or without exercise

Male C57BL/6 J mice, aged 4 weeks, were housed in optimal conditions (21 ± 1°C, 50% humidity, 12 h light/dark cycle) with unrestricted access to food and water. Following a one-week acclimatization period, the mice were divided into three groups: a normal fat diet (NFD) group, a high-fat diet (HFD) group (60% energy from lipids), and an HFD plus exercise group. For the exercise regimen, mice underwent a two-day adaptation period with 10 min of daily swimming to minimize stress. After adaptation, the swimming duration was set to 30 min daily.

### Establishment and treatment of the needle-induced IVDD mouse model

After 10 weeks of HDF, the mice were divided into three groups: sham group, IVDD group, and IVDD + exercise group. Mice were subject to needle surgery to in-duce IVDD as previously described (Han et al., 2025). Briefly, under isoflurane anesthesia, mice were positioned prone, and the target coccygeal intervertebral disk (IVD) segment was identified via palpation. Following disinfection, a small sagittal incision was made, and a 25G sterile needle was inserted 1.5 mm into the IVD tissue. The needle was then rotated 180° axially and held for 30 s. Adjacent IVD segments remained untouched as controls. IVD tissues were collected 4 weeks post-surgery. In the IVDD + exercise group, mice underwent daily 30-min swimming sessions.

### Histology staining

For liver tissue analysis, the harvested livers were dehydrated through a series of progressively graded alcohol solutions over 48 h and subsequently embedded in paraffin. Liver sections, 5 μm thick, were prepared using a semi-automated rotary microtome and stained with hematoxylin and eosin (H&E). Additionally, frozen liver sections, 10 μm thick, were stained with Oil Red O (Solarbio Life Science) for further examination. For immunofluorescence (IF) staining of F4/80 (GB113373, Servicebio) in mouse liver tissue, the prepared liver tissue sections were initially blocked using an IF staining blocking buffer (G2010, ServiceBio) containing Triton X-100 (G1204, ServiceBio). The sections were then incubated with primary antibodies against F4/80, a specific marker for macrophages, at 4°C overnight. Following this, the sections were treated with CY3-labeled Donkey Anti-Goat IgG (GB21404, Servicebio) and counterstained with DAPI (G1012, Servicebio) to visualize nuclei. Finally, the stained sections were examined under a confocal microscope, and the images were analyzed using ImageJ software.

For mouse IVD tissues, the tissues were collected and then fixed with 4% paraformaldehyde for 2 days, followed by decalcification in 14% ethylenediaminetetraacetic acid solution for 2 weeks, dehydrated, paraffin embedded and section at 5 μm prior to staining. The sections were stained with H&E as well as safranin O and fast green (SOFG) to facilitate the analysis of morphological changes in the IVD tissues. To quantitatively evaluate IVDD, we employed a new-proposed histological scoring system that assesses the sub-features of IVD (Melgoza et al., 2021). To ensure objectivity, histological scoring was performed by two independent researchers who were blinded to the group assignments. For immunohistochemical (IHC) staining, antigen retrieval was performed using a 0.1 mol·L^−1^ citrate buffer solution (pH 6.0). Subsequently, the sections were blocked with a peroxidase-blocking solution and normal horse serum to minimize non-specific binding. Following this, the sections were incubated with primary antibodies at 4°C overnight. After incubation, the sections were treated with biotinylated IgG and streptavidin-horseradish peroxidase to amplify the signal, and immunoreactivity was visualized using the DAB Peroxidase Substrate Kit. Finally, the sections were counterstained with hematoxylin to provide contrast and then mounted for microscopic analysis. The primary antibodies utilized in this study included Aggrecan (ACAN, DF7561, Affinity) and MMP3 (340,612, ZenBio).

### Quantitative real-time polymerase chain reaction (qRT-PCR)

Total RNA was isolated from mouse liver or IVD tissues using Trizol reagent (Invitrogen, Carlsbad, CA, United States). Subsequently, reverse transcription and amplification were carried out using the HiScript^®^ III RT SuperMix for qPCR Kit (R323-01, Vazyme) on a Real-Time PCR system (Applied Biosystems, Foster City, United States). The relative expression levels of target genes were quantified using SYBR qPCR Master Mix (Q711-02, Vazyme) and calculated using the 2^^–ΔΔCt^ method, with GAPDH serving as the internal reference gene for normalization. All experiments were conducted in triplicate to ensure reproducibility and accuracy.

## Results

### Analysis of DEGs for IVDD and NAFLD

**Figure 1:**
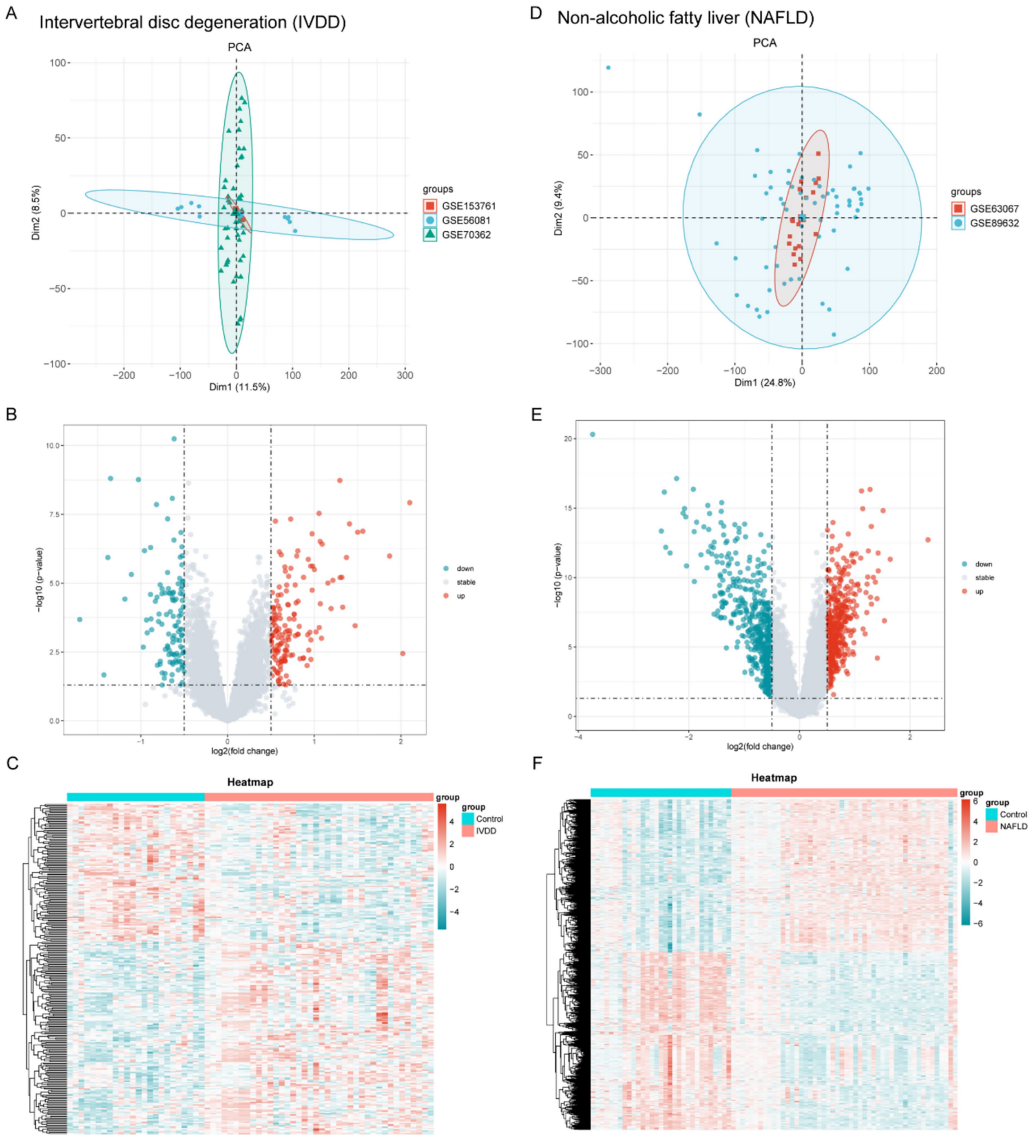
Batch effect correction and discovery of DEGs in IVDD and NAFLD. **(A)** PCA plots depict the expression patterns within three IVDD datasets after removing the batch effects. **(B,C)** Volcano plot and heatmap illustrating DEGs in the IVDD group. **(D)** PCA plots depict the expression patterns within two NAFLD datasets after removing the batch effects. **(E,F)** Volcano plot and heatmap illustrating DEGs in the NAFLD group. DEG, differentially expressed genes; NAFLD, non-alcoholic fatty liver disease; IVDD, Intervertebral disk degeneration; PCA, principal component analysis.

Upon acquisition of the datasets, we initially assessed the batch effects across them before proceeding with the biological inflammation analysis. Subsequently, we used the “sva” R package to eliminate batch effects in IVDD and NAFLD datasets, respectively (Figures 1A,D). DEGs were found using the “limma” R package for both IVDD and NAFLD (Figures 1B–F). For IVDD, there were 266 DEGs, comprising 155 upregulated and 111 down-regulated genes. In terms of NAFLD, 970 DEGs were found, including 448 upregulated and 522 down-regulated genes. Volcano plots and heat maps displayed all DEGs in the IVDD (Figures 1B,C) and NAFLD groups (Figures 1E,F).

### Identification of the critical gene modules through WGCNA

**Figure 2:**
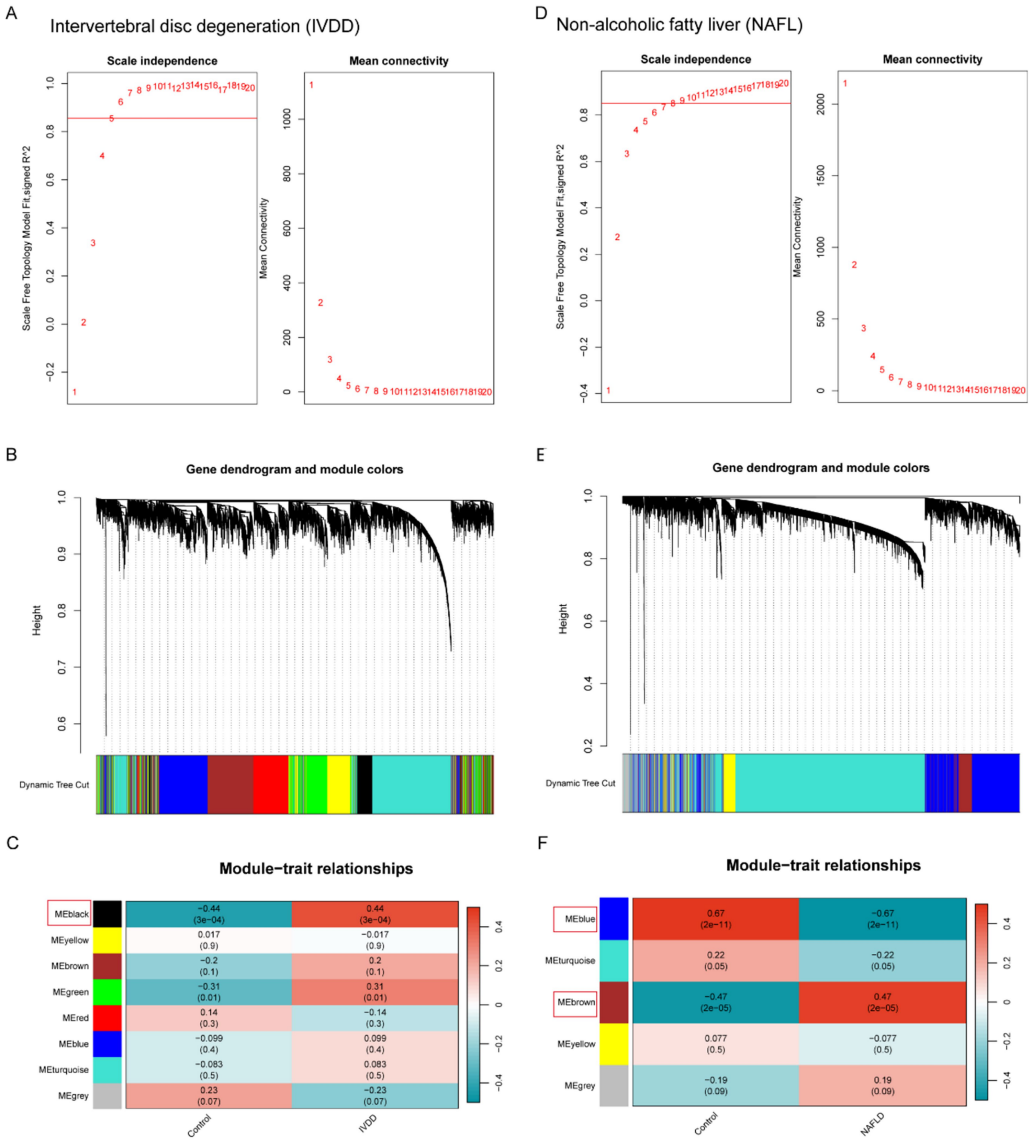
Weighted gene co-expression network analysis of IVDD and NAFLD. **(A)** Identifying the soft-threshold power in IVDD. **(B)** Cluster dendrogram displaying highly connected genes in key modules associated with IVDD. **(C)** Interconnections among modules and traits in IVDD. Correlation coefficients and *p*-values are incorporated in each cell. **(D)** Identifying the soft-threshold power in IVDD. **(E)** Cluster dendrogram displaying highly connected genes in key modules associated with IVDD. **(F)** Interconnections among modules and traits in IVDD. Correlation coefficients and *p*-values are incorporated in each cell. WGCNA, weighted gene co-expression network analysis; NAFLD, non-alcoholic fatty liver disease; IVDD, Intervertebral disk degeneration.

An analysis of DEGs between the two disease groups was conducted in conjunction with WGCNA in order to explore the potential correlation between these two diseases and key genes. A co-expression network was built utilizing the soft-thresholding method, with the parameter *β* being essential for preserving a scale-free topology within the network. Given that biological networks constructed from gene expression data are often scale-free, a fit index greater than the threshold 0.85 in the IVDD group suggested the presence of scale-free topology, with the soft-thresholding power *β* set at 5 (Figure 2A). We then created an adjacency matrix by applying the adjacency function and established hierarchical clustering based on the TOM dissimilarity metric (Figure 2B). Modules that exhibited a *p*-value below 0.05 were deemed significant and considered as key modules. According to Figure 2C, eight modules were detected, among which the MEblack module showed the most pronounced negative correlation and contained a specific number of genes (Figures 2B,C). Likewise, WGCNA was used to the NAFLD group, determining that *β* = 8 was the optimal value for soft power (Figure 2D). Among these modules, the MEblue module demonstrated a strong positive correlation, whereas the MEbrown module exhibited a robust negative correlation. Together, they encompassed a specific number of genes (as shown in Figures 2E,F). These genes from the key modules identified across both groups could potentially serve as candidate markers.

### Investigation of shared genes for IVDD and NAFLD

We conducted an intersection analysis between the DEGs and the genes identified from WGCNA in order to investigate the common pathogenesis of IVDD and NAFLD. A total of six genes (ME1, HAS2, ADRS2, RASD1, PLCD1, and FICD) were highlighted. We hypothesize that these six genes could be instrumental in the development of both IVDD and NAFLD, possibly indicating a shared mechanism (Supplementary Figure S2).

### Identification of shared diagnostic biomarkers via machine learning algorithms for IVDD and NAFLD

**Figure 3:**
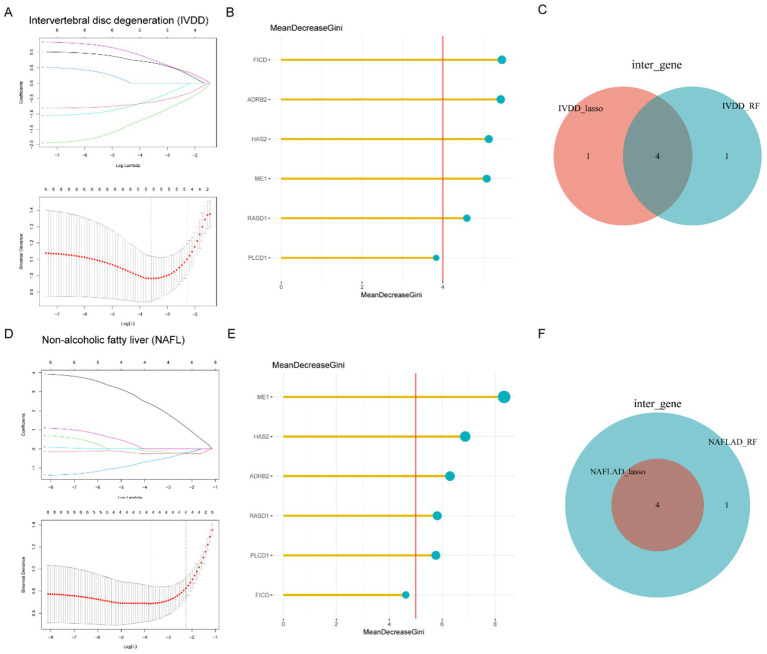
Identification of potential diagnostic genes for IVDD and NAFLD through the application of machine learning algorithms. **(A)** The LASSO logistic regression algorithm was used to identify the minimum and lambda values for diagnostic biomarkers. **(B)** Top six genes in IVDD identified for their discriminatory power in the RF algorithm. **(C)** Venn diagram illustrating the four candidate diagnostic genes identified by the LASSO and RF algorithms. **(D)** The LASSO logistic regression algorithm was used to determine the minimum values and lambda coefficients for diagnostic biomarkers. **(E)** Top six genes in the RF algorithm with discriminative efficacy for NAFLD. **(F)** Venn diagram illustrating four candidate diagnostic genes in NAFLD by the intersection of results from two algorithms. IVDD, Intervertebral disk degeneration; NAFLD, Non-alcoholic fatty liver disease; LASSO, least absolute shrinkage and selection operator; RF, random forest.

Using the six common genes mentioned above, we applied LASSO and RF to pinpoint diagnostic gene targets. In the IVDD cohort, LASSO regression singled out four genes with significant diagnostic influence (Figure 3A). A more nuanced selection of biomarkers was achieved by prioritizing these six genes based on their importance scores, with a threshold at the top two, resulting in five biomarkers being identified (Figure 3B). By overlapping the outcomes from LASSO and RF, we established a panel of four shared biomarkers (ME1, HAS2, ADRB2, and PLCD1) for the IVDD group (Figure 3C). In parallel, LASSO identified five distinct genes in the NAFLD group (Figure 3D). Figure 3E highlights six genes with importance scores exceeding 4 based on RF results. The intersected biomarkers (ME1, HAS2, ADRB2, and RASD1) identified by both algorithms for NAFLD are depicted in Figure 3F.

### Single-gene KEGG of diagnostic genes

To gain a thorough insight into the connection between IVDD and NAFLD, we intersected the results of machine learning from both groups, revealing three common diagnostic genes (ME1, HAS2, and ADRB2) (Supplementary Figure S3A). The performance of these three shared diagnostic genes in terms of prediction and discrimination was evaluated through the analysis of their expression alternations and ROC analyses. We first investigated the levels of ME1, HAS2, and ADRB2 in IVDD and NAFLD cohorts. As depicted in Supplementary Figure S3, ME1 displayed elevated expression in both IVDD and NAFLD groups, whereas HAS2 and ADRB2 exhibited decreased expression in both groups (Supplementary Figures S3B,C).

To investigate the role of ME1, HAS2, and ADRB2 in IVDD and NAFLD, KEGG analysis was conducted. In the context of IVDD, KEGG analysis indicated the diverse effects of ME1, identifying pathways associated with cell growth regulation (PI3K-AKT, TGFβ, AMPK, Wnt, and Hippo signaling), inflammation (TNF, NFκB, AGE-RAGE, and IL-17 signaling), immune responses (Malaria), cell death (NOD-like receptor signaling), and ECM homeostasis (ECM-receptor interaction and focal adhesion) (Supplementary Figure S4A). For HAS1 in IVDD, enrichment was observed in fundamental signaling pathways such as “PI3K-AKT signaling pathway,” “ECM-receptor interaction,” and “focal adhesion,” and “AGE-RAGE signaling” (Supplementary Figure S4B). As for ADRB2 in IVDD, KEGG analysis suggested this gene was mainly correlated with oxidative stress (reactive oxygen species), inflammation (TNF and IL-17 signaling pathways), immune responses (Hepatitis B, osteoclast differentiation, and complement and coagulation cascades), and metabolic pathways (alanine, arginine and proline, and steroid hormone metabolism) (Supplementary Figure S4C).

In the case of NAFLD, a KEGG analysis demonstrated the varied impacts of ME1, revealing its involvement in key metabolic pathways such as the “PPAR signaling pathway,” “fatty acid metabolism,” and “*α*-linolenic acid metabolism” (Supplementary Figure S4D). In NAFLD, the enrichment pathways associated with HAS1 encompassed cell growth regulation (PI3K-AKT and AMPK signaling), inflammation (TNF, AGE-RAGE, and IL-17 signaling), and immune responses (Hepatitis B and malaria) (Supplementary Figure S4E). As for ADRB2 in NAFLD, KEGG analysis demonstrated that ADRB2 was primarily associated with cell growth regulation (PI3K-AKT signaling), inflammation (TNF, AGE-RAGE, and IL-17 signaling pathways), immune responses (Th17 cell differentiation and Influenza A), and cellular senescence (p53 signaling) (Supplementary Figure S4F).

### Analysis of immune cell infiltration

**Figure 4:**
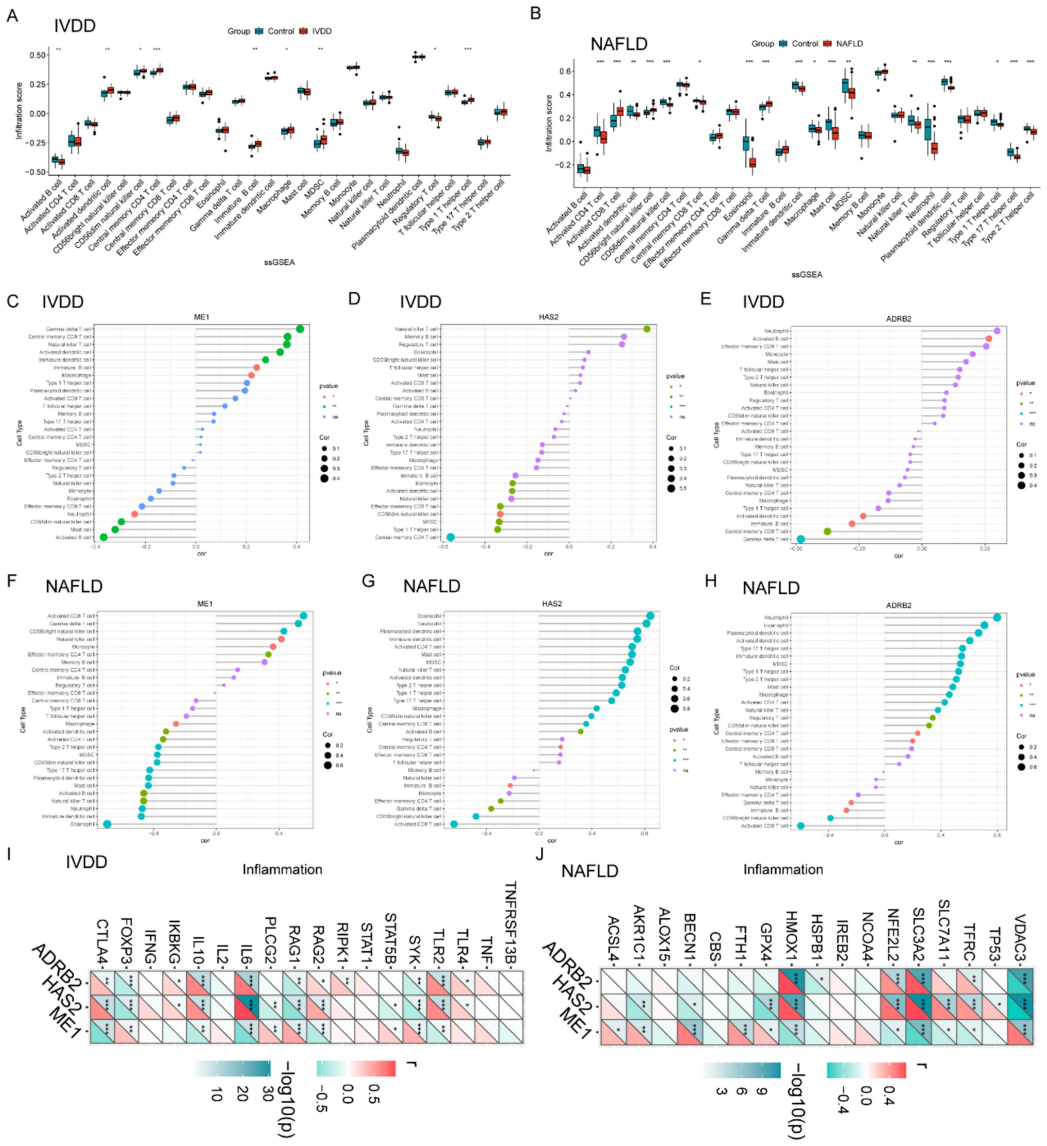
Intervertebral disk degeneration and NAFLD immune cell infiltration analysis. **(A)** Violin diagram indicating the comparison of 28 types of immune cells between the IVDD and control groups. **(B)** Violin diagram indicating the comparison of 28 types of immune cells between the NAFLD and control groups. **(C–E)** Correlation between ME1, HAS2, and ADRB2 expression and immune cells in the IVDD group. **(F–H)** Relationship between ME1, HAS2, and ADRB2 expression and immune cells in the NAFLD group. **(I)** Correlation of hub genes in IVDD with immune factors. **(J)** Correlation of hub genes in NAFLD with immune factors. *p* < 0.05 was highlighted. IVDD, Intervertebral disk degeneration; NAFLD, non-alcoholic fatty liver disease.

Box plots were used to compare immune cell infiltration between IVDD and control groups (Figure 4A). The results indicated significant differences in the distribution of various immune cell types between the two groups, including activated B cells, activated dendritic cells, natural killer cells, central memory CD8 T cells, immature B cells, macrophages, myeloid-derived suppressor cells (MDSCs), regulatory T cells, and type 1T helper cells (Figure 4A). For NAFLD, The immune cell infiltration analysis showed statistically significant differences in the distribution of various immune cell types between the two groups, including activated CD4^+^ T cells, activated CD8^+^ T cells, activated dendritic cells, natural killer cells, central memory CD8 T cells, Eosinophil, γδT cell, immature dendritic cells, macrophages, mast cells, myeloid-derived suppressor cells (MDSCs), neutrophil, type 2T helper cells, type 17T helper cells, and type 1T helper cells (Figure 4B).

Further correlation analyses between hub genes (ME1, HAS2, and ADRB2) expression and immune cell proportions in IVDD revealed evident correlations (Figures 4C–E). ME1 was positively correlated with γδT cell, central memory CD8 T cells, natural killer T cell, immature B cells, activated dendritic cells, and macrophages while negatively correlating with activated B cells, mast cells, CD56dim natural killer cell, and neutrophil (Figure 4C). For HAS2, this gene expression was positively correlated with natural killer T cell, while negatively correlating with central memory CD4 T cells, type 1T helper cells, MDSC, CD56dim natural killer cell, effector memory CD8 T cell, activated dendritic cells, and monocyte (Figure 4D). ARDB2 was negatively correlated with γδT cell, central memory CD8 T cells, immature B cells, and activated dendritic cells, while positively correlating with activated B cells (Figure 4E).

However, in NAFLD, ME1 was positively correlated with activated CD8 T cell, γδT cell, central memory CD8 T cells, CD56dim natural killer cell, natural killer T cell, monocyte, etc., while negatively correlating with Eosinophil, immature dendritic cell, neutrophil, natural killer cell, activated B cells, mast, macrophage, etc. (Figure 4F). HAS2 was negatively correlated with activated CD8 T cell, CD56bright natural killer cell, γδT cell, effector memory CD4 T cells, monocyte, etc., but positively correlated with eosinophil, neutrophil, plasmacytoid dendritic cell, activated CD4 T cell, etc. (Figure 4G). For ADRB2, this gene expression was positively correlated with eosinophil, neutrophil, plasmacytoid dendritic cell, activated CD4 T cell, etc., while negatively correlating with activated CD8 T cells, CD56bright natural killer cell, immature B cell, γδT cell, etc. (Figure 4H). Subsequently, we retrieved immunity-related gene sets from the GeneCards database^2^ and extracted the expression levels of the top 20 genes. Furthermore, we revealed the close relevance between the three hub genes and inflammatory genes in the context of IVDD and NAFLD (Figures 4I,J).

These findings highlight the potential importance of the three key genes, ME1, HAS2, and ADRB2, in regulating immune responses associated with IVDD and NAFLD, suggesting a comparable immunological profile shared by both conditions.

### Examination of hub gene expression and molecular function in single cell level

**Figure 5:**
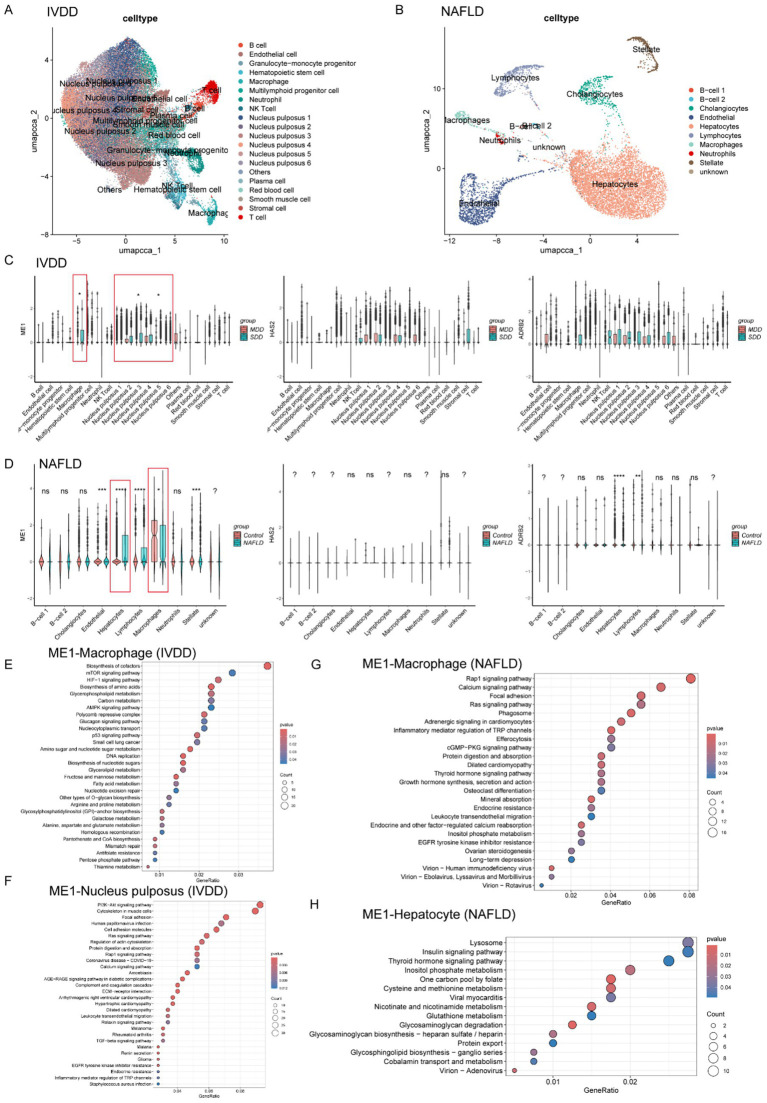
Expression profiles and function enrichment of hub genes in single cell level for IVDD and NAFLD. **(A)** UMAP visualizing human IVD cells as different cell clusters after unsupervised clustering. Each plot indicated one single cell and colored based on different cell subclusters. **(B)** UMAP visualizing human liver cells as different cell clusters after unsupervised clustering. Each plot indicated one single cell and colored based on different cell subclusters. **(C)** The relative expression and distribution of hub genes across human IVD cell clusters in mild degenerated and severely degenerated IVD tissue, respectively. **(D)** The relative expression and distribution of hub genes across human liver cell clusters in control and NAFLD group, respectively. **(E,F)** KEGG analysis of ME1 for macrophages and NPCs, respectively. **(G,H)** KEGG analysis of ME1 for macrophages and hepatocytes, respectively. IVDD, Intervertebral disk degeneration; NAFLD, non-alcoholic fatty liver disease; KEGG: Kyoto Encyclopedia of Genes and Genomes.

We acquired the single-cell datasets for IVDD (GSE244889) and NAFLD (GSE202379) separately. The method has been reported in our previous study (Zhang et al., 2024). The Seurat package facilitated the processing of scRNA-seq data. Initially, low-quality cells were excluded, leaving only high-quality cells which were then normalized and scaled through the “NormalizeData” and “ScaleData.” PCA was performed on the top 2,000 variable genes, and subsequently, the 30 most statistically significant principal components were selected for further cluster analysis. To mitigate batch effects, the “IntegratedLayers” function was employed. Then, cell clustering was achieved through Uniform Manifold Approximation and Projection (UMAP), and cluster annotation was performed using the R package SingleR. For IVDD, the cells were categorized into a total of 20 clusters, encompassing NPC clusters 1–6, B cells, endothelial cells, granulocyte-monocyte progenitors, hematopoietic stem cells, macrophages, multilymphoid progenitor cells, neutrophils, NK T cells, plasma cells, red blood cells, smooth muscle cells, T cells, and other types (Figure 5A). In the case of NAFLD, the cell clusters primarily consisted of B cell subsets 1–2, cholangiocytes, endothelial cells, hepatocytes, lymphocytes, macrophages, neutrophils, stellate cells, and some uncharacterized cells (as Figure 5B). All the cell clusters were annotated using the CellMarker website.^3^

Subsequently, we analyzed the expression levels of hub genes (ME1, HAS2, and ADRB2) of all the cell clusters in IVDD and NAFLD, respectively. In IVDD, the ME1 and ADRB2 genes are predominantly expressed in macrophages and NPC clusters, while the HAS2 gene is mainly expressed in NK T cells as well as NPC clusters (Figure 5C). It’s worth noting that only the ME1 expression level in macrophages and NPC clusters exhibits a statistically significant difference between mildly and severely degenerated IVD tissues (Figure 5C). In the context of NAFLD, the ME1 gene is broadly expressed across all cell clusters within the liver, whereas the HAS2 gene is primarily expressed in stellate cells (Figure 5D). The ADRB2 gene can be detected in most liver cell clusters, excluding B cells (Figure 5D). Importantly, statistical differences in ME1 expression levels were only observed between macrophages and hepatocytes in the control group and the NAFLD group (Figure 5D).

Given the aforementioned results, we chose to focus on the molecular function of ME1 because of its distinctive expression patterns in macrophages, NPCs, and hepatocytes. To better investigate the role of ME1 in IVDD and NAFLD, we categorized the macrophages, NPCs, and hepatocytes into two groups based on ME1 expression. In IVDD, ME1 correlated mainly with metabolic pathways in macrophages, such as mTOR signaling pathway, HIF-1 signaling pathway, AMPK signaling pathway, carbon metabolism, and biosynthesis of amino acids (Figure 5E). As for NPCs, ME1 participated in the regulation of PI3K-Akt signaling pathway, cytoskeleton, and focal adhesion, which was indispensable for the survival and proliferation of NPCs and IVD hemostasis (Risbud et al., 2010; Gong et al., 2023) (Figure 5F). In the context of NAFLD, ME1 in macrophages was mainly involved in the processes, such as Rap1 signaling pathway, calcium signaling pathway, focal adhesion, phagosome, and efferocytosis (Figure 5G). In hepatocytes, ME1 was linked to lysosomes and insulin signaling pathway, as well as various metabolic processes, including the inositol phosphate signaling pathway, one-carbon metabolism via folate, and cysteine and methionine metabolism (Figure 5H).

**Figure 6:**
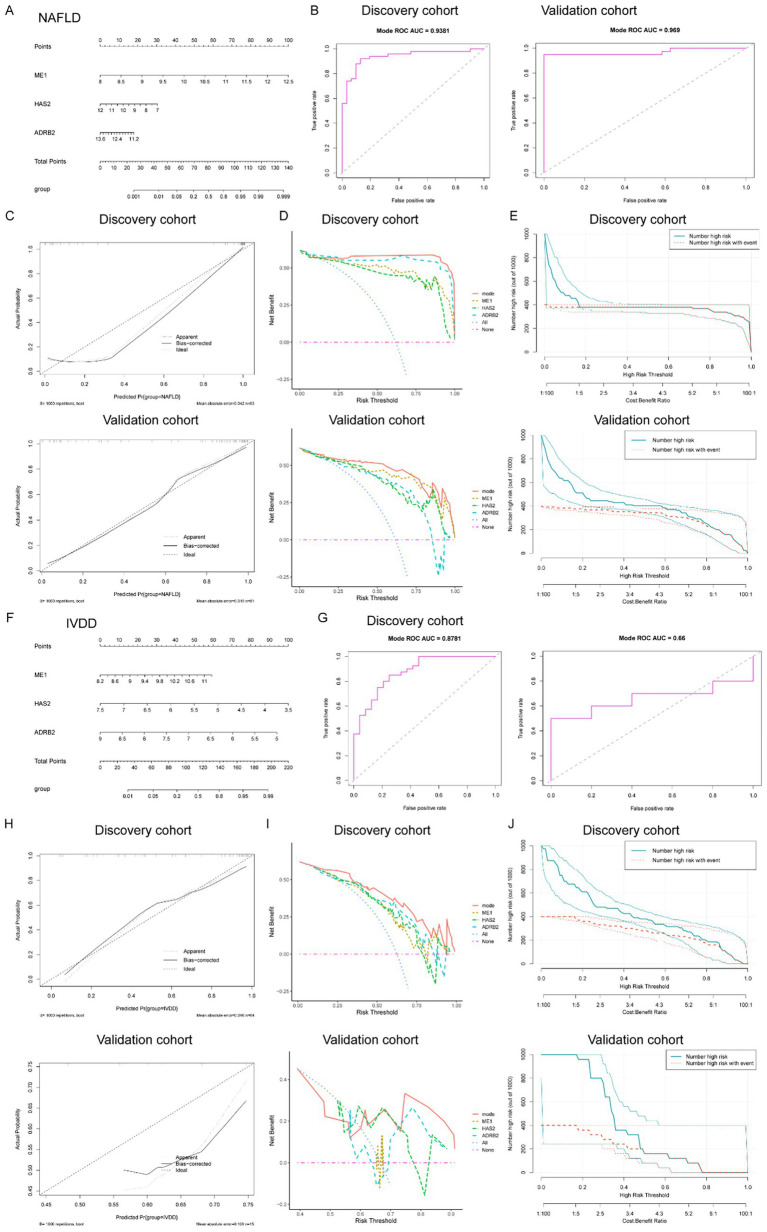
Determination of the diagnostic genes and establishment of the diagnostic nomogram mode in common between IVDD and NAFLD. **(A)** Nomogram constructed based on the diagnostic biomarkers for risk prediction of IVDD. **(B)** ROC curve of ME1, HAS2, and ADRB2 in the training set (combined datasets) and the validation set for NAFLD. **(C)** Calibration curve, DCA curve **(D)**, and clinical impact curve **(E)** for assessing the performance of the nomogram’ in both the training (combined datasets) and validation sets for NAFLD. **(F)** Nomogram constructed based on the diagnostic biomarkers for risk prediction of IVDD. **(G)** ROC curve of ME1, HAS2, and ADRB2 in the training set (combined datasets) and the validation set for IVDD. **(H)** Calibration curve, DCA curve **(I)**, and clinical impact curve **(J)** for assessing the performance of the nomogram’ in both the training (combined datasets) and validation sets for IVDD. IVDD, Intervertebral disk degeneration; NAFLD, non-alcoholic fatty liver disease; ROC, receiver operating characteristic; DCA, decision curve analysis.

Collectively, the results above suggested the vital effects of these three hub genes on either IVDD or NAFLD.

Diagnostic significance of key diagnostic biomarkers and the establishment of a diagnostic nomogram model for IVDD and NAFLD.

To enhance the diagnostic and predictive efficacy, a diagnostic nomogram model for NAFLD was developed, utilizing the three key genes: ME1, HAS2, and ADRB2, as depicted in Figure 6A. Each risk factor was assigned a score, and the sum of these scores was used as an indicator to evaluate the likelihood of NAFLD development in patients. Within the NAFLD cohort, ME1 (with an AUC of 0.9194), HAS2 (AUC = 0.08806), and ADRB2 (AUC = 0.08316) exhibited dependable predictive abilities. ROC curve showed that the AUC for the risk score in the established model was 0.9381 (Figure 4B). Similarly, when the validation dataset was applied to plot the ROC curve, the AUC of the risk score reached 0.969 (Figure 6B). The calibration curves, in the training and validation sets, as depicted in Figure 4C, closely matched the standard curve, demonstrating the nomogram’s high predictive accuracy for NAFLD (Figure 6C). Furthermore, the DCA curve and CIC (Figures 6D,E) indicated the strong performance of the risk model in both the training and validation sets.

Analogously, we constructed a diagnostic nomogram model for IVDD based on the three hub genes, ME1, HAS2, and ADRB2 (Figure 6F). We then evaluated the specificity and sensitivity of the three hub genes in diagnosing IVDD. The results for the IVDD biomarkers were satisfactory, with ME1 (area under the curve [AUC] = 0.7448), HAS2 (AUC = 0.7604), and ADRB2 (AUC = 0.7948) exhibiting robust predictive performance. ROC curve showed that the AUC for the risk score in the established model was 0.8781 (Figure 6G). Similarly, when the validation dataset was applied to plot the ROC curve, the AUC of the risk score reached 0.66 (Figure 6G). The calibration curves, in both the training and validation sets, also closely aligned with the standard curve, demonstrated the nomogram’s high predictive accuracy for IVDD (Figure 6H). Moreover, the DCA curve and clinical impact curves (CIC) suggested the strong performance of the risk model in both the training and validation sets (Figures 6I,J).

In conclusion, these findings demonstrate the robust predictive power of the risk-score model and highlight the crucial role that the three diagnostic biomarkers play in the progression of IVDD or NAFLD.

### HFD resulted in NAFLD and aggravated needle-induced IVDD *in vivo*

**Figure 7:**
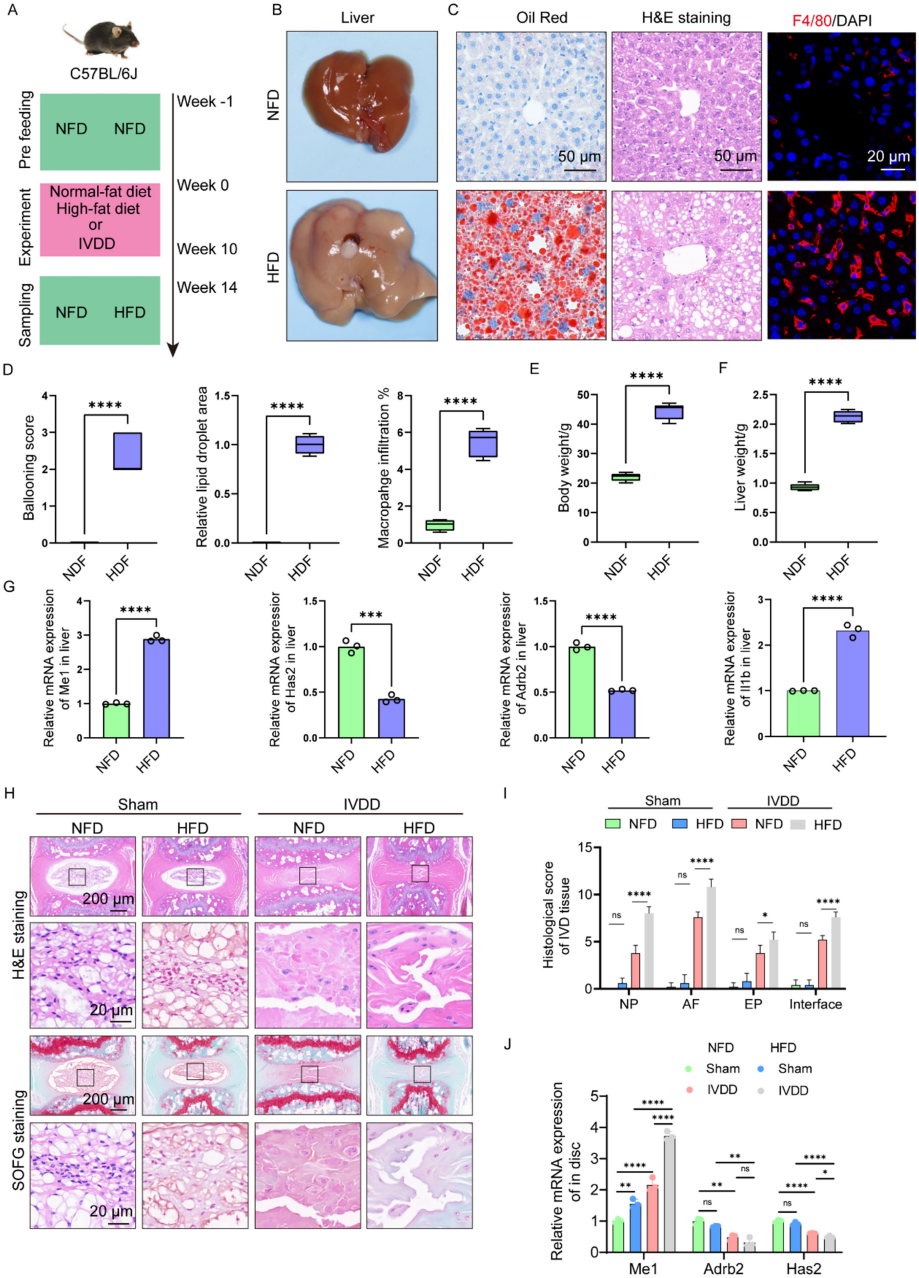
High-fat diet resulted in NAFLD and aggravated needle-induced IVDD *in vivo*. **(A)** Illustration of the experiment design to investigate the effects of NAFLD on IVDD. **(B)** Liver image from NFD and HFD mice, respectively. **(C)** Histology analysis including H&E (scale: 50 μm), Oil Red (scale: 50 μm), and F4/80 IHC staining (scale: 20 μm). **(D)** Ballooning score, lipid droplet area, and inflammation score of liver from NFD and HFD mice, respectively (*n* = 5). **(E)** Body weight from NFD and HFD mice, respectively (*n* = 5). **(F)** Liver weight from NFD and HFD mice, respectively (*n* = 5). **(G)** Relative mRNA levels of Me1, Has2, Adrb2 and Il1b in liver from NFD and HFD mice, respectively (*n* = 3). **(H)** H&E and SOFG staining of the IVD tissue from mice in different groups. **(I)** Histological score of the IVD tissue from mice in different groups (*n* = 5). **(J)** Relative mRNA levels of Me1, Has2, Adrb2 and Il1b in IVD tissue from mice in different groups (*n* = 3). *p < 0.05, **p < 0.01, ***p < 0.001, and ****p < 0.0001.

To further confirm the relationship between IVDD and NAFLD, we firstly established NAFDL mouse model. The mice were administrated with NFD for a week, and then fed with HFD in the following 10 weeks to induce NAFLD model. Next, the caudal disk of the mice was punctured using a 25G needle and continue, followed by continued HFD feeding for an additional 4 weeks (Figure 7A). Finally, the liver and IVD tissue were collected for further experiments. The results showed that HFD significantly induced liver enlargement, a soft texture, a light-yellow color, and a greasy appearance (Figure 7B). Histological analyses indicated that HFD resulted in significant hepatic steatosis and inflammatory infiltration (Figures 7C,D). Unsurprisingly, HFD induced the increases in body weight and liver weight (Figures 7E,F). These results above suggested the successful establishment of NAFDL model. We evaluated the gene expression of Me1, Adrb2, Has2, and Il1b in mouse liver, and found that mice with NAFLD expressed higher level of Me1 and Il1b, and lower level of Adrb2 and Has2, consistent with the results of bioinformatics analysis above (Figure 7G).

Then, we evaluated the IVD tissue histologically. H&E staining and histological score demonstrated that HFD slightly affected the sub-structures of IVD in sham group, despite no significant difference (Figures 7H,I). However, in the needle-induced IVDD model, the IVD of mice with HFD group showed more severe degeneration-related changes, including worse cellularity, morphology, matrix organization in NP, EP, and AF, respectively, compared with those of mice with NFD group (Figures 7H,I). In addition, NP-AF boundary, NP-EP boundary, and the AF lamella disruption into the EP became more blurred in the IVD of mice with HFD group (Figures 7H,I). We also examined the expression of Me1, Adrb2, Has2, and Il1b in mouse IVD tissue, and found that mice with NAFLD expressed higher level of Me1 and Il1b, and lower level of Adrb2 and Has2, consistent with the results of liver (Figure 7J).

Collectively, we deduced that HFD could result in NAFLD and aggravated needle-induced IVDD *in vivo*, and the hub genes (Me1, Adrb2, and Has2) may play critical role in regulating these pathological changes.

### Exercise alleviated the progression of IVDD concurrently with NAFLD

**Figure 8:**
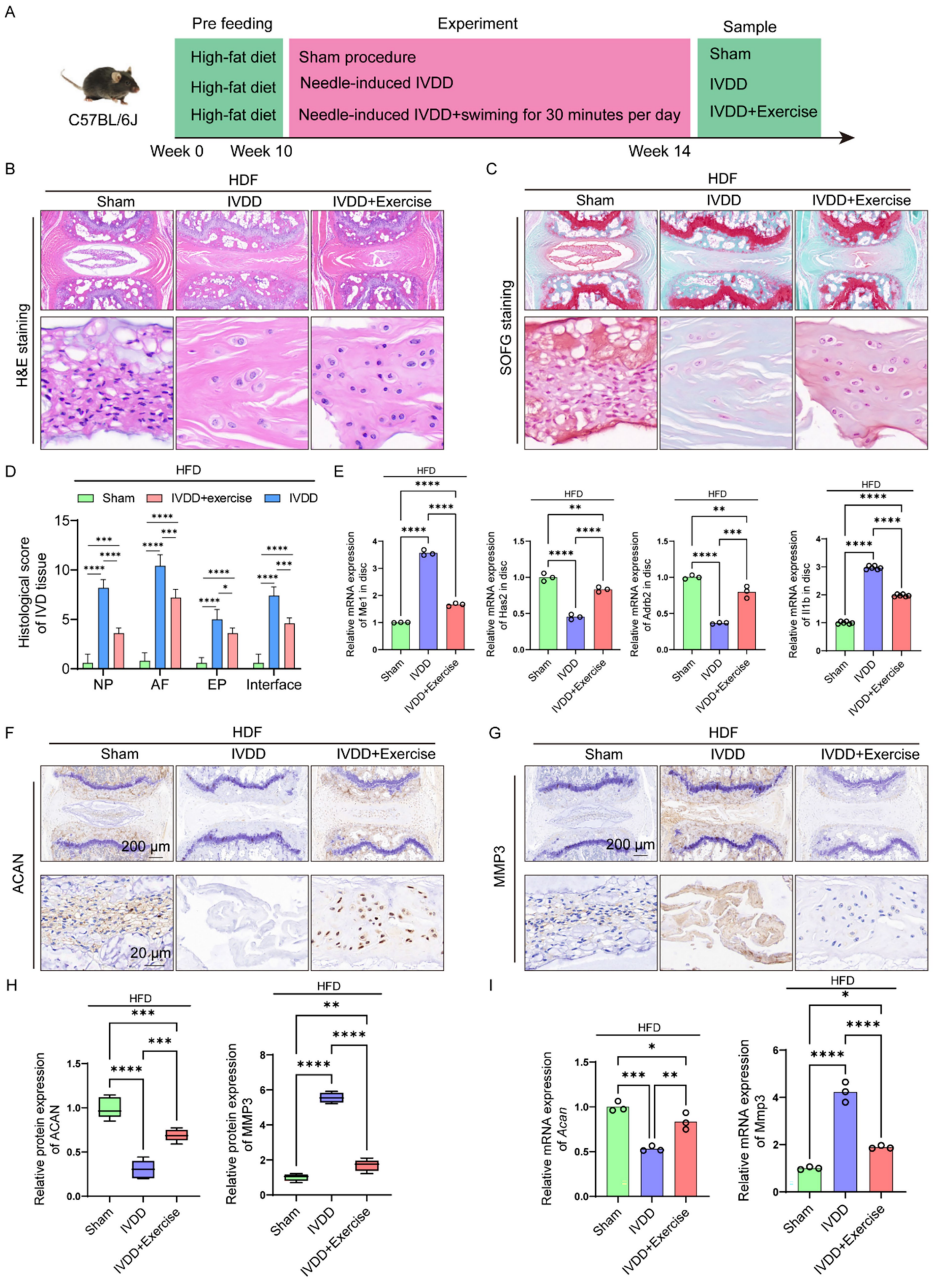
Exercise alleviated the progression of IVDD concurrently with NAFLD. **(A)** Illustration of the experiment design to investigate the effects of alleviating NAFLD via swimming on IVDD. **(B)** H&E staining of the IVD tissue from mice in different groups. **(C)** SOFG staining of the IVD tissue from mice in different groups. **(D)** Histological score of the IVD tissue from mice in different groups (*n* = 5). **(E)** Relative mRNA levels of Me1, Has2, Adrb2 and Il1b in IVD tissue from mice in different groups (*n* = 3). **(F,G)** IHC staining for ACAN and MMP3 in the IVD tissue from mice in different groups. **(H)** Quantitation results of IHC analyses for ACAN and MMP3 in the IVD tissue from mice in different groups (*n* = 3). **(I)** Relative mRNA levels of Acan and Mmp3 in IVD tissue from mice in different groups (*n* = 3). *p < 0.05, **p < 0.01, ***p < 0.001, and ****p < 0.0001.

Physical exercise is beneficial to human health and is related to reducing the risk of multiple diseases, including NAFLD (Zhou et al., 2022). To further elucidate the protective effects of exercise-mediated NAFLD amelioration on IVDD progression, we initially established a high-fat diet (HFD)-induced NAFLD murine model. Following NAFLD induction, the mice underwent IVDD surgery and were subsequently subjected to swimming-based exercise intervention (Figure 8A). Histopathological analyses through H&E and SOFG staining revealed that NAFLD exacerbated IVDD severity *in vivo*, while exercise intervention significantly attenuated these pathological changes, restoring the intervertebral disk structure to a relatively preserved state (Figures 8B–D). Molecular analysis demonstrated that exercise intervention in NAFLD mice led to downregulation of Me1 and Il1b expression and upregulation of Adrb2 and Has2 within the intervertebral disk tissue (Figure 8E). These results collectively indicate that exercise intervention effectively mitigated IVDD progression in NAFLD-afflicted mice, suggesting a potential therapeutic strategy for managing IVDD in the context of metabolic disorders.

Subsequently, we investigated the therapeutic potential of exercise-mediated NAFLD amelioration on IVD histological alterations. IHC analysis revealed that NAFLD-afflicted mice exhibited significantly reduced ACAN protein expression and elevated MMP3 levels, providing further evidence that NAFLD exacerbates IVDD progression (Figures 8F–H). Notably, exercise intervention substantially attenuated these pathological changes in IVD tissue (Figures 8F–H). Complementary RT-qPCR analysis demonstrated consistent alterations in the gene expression patterns of Acan and Mmp3, corroborating the protein-level findings (Figure 8I). These results collectively suggest that exercise intervention effectively counteracts the detrimental effects of NAFLD on IVD tissue homeostasis.

Taken together, exercise could alleviate the progression of IVDD concurrently diagnosed with NAFLD.

## Discussion

A recent review has clarified that, apart from genetic factors, IVDD frequently co-occurs in individuals with obesity or type 2 Diabetes Mellitus, two conditions that constitute metabolic syndrome (Francisco et al., 2022). In our recent study, we revealed that obesity increased the susceptibility to IVDD via inducing ECM disfunction and angiogenesis (Han et al., 2025). Currently, NAFLD is widely recognized as a chronic disorders, also associated with obesity (Leoni et al., 2018; Bessone et al., 2019). Recent research indicates a robust link between IVDD and NAFLD in the past few years (Zhou et al., 2024; Francisco et al., 2022; Fu et al., 2024). However, the mechanisms underlying these conditions remain elusive. In this present study, the combination of DEGs and WGCNA revealed six genes (ME1, HAS2, ADRS2, RASD1, PLCD1, and FICD) related to IVDD and NAFLD. The final alignment of results from machine learning identified three shared diagnostic biomarkers: ME1, HSA2, and ADRB2. Notably, Sc-RNA seq revealed that ME1 showed the most significant expression changes and exerted wide role in regulating the function of macrophages, nucleus pulposus, and hepatocytes. Finally, we proved that NAFLD could aggravate the progression of IVDD *in vivo*, and alleviating NAFLD via swimming showed protective effects on IVDD in the context of HFD. Furthermore, we developed diagnostic nomogram models for IVDD and NAFLD, which demonstrated high diagnostic accuracy and good practical significance in clinical settings. In this study, we constructed separate nomograms for NAFLD and IVDD, with the following concerns. Firstly, while the shared genes (ME1, HAS2, ADRB2) highlight common pathogenic pathways, NAFLD and IVDD are clinically distinct entities. Separate nomograms allow for disease-specific risk stratification, which is more actionable for clinicians, such as hepatologists diagnosing NAFLD and orthopedic surgeons managing IVDD. Secondly, the models demonstrate that the same genes can effectively predict both diseases, reinforcing their biological relevance. This aligns with our central hypothesis, despite tissue-specific differences, these genes contribute to shared mechanisms, such as inflammation or metabolic dysregulation. The nomograms, though developed separately, underscore the translational relevance of ME1, HAS2, and ADRB2. Their predictive accuracy aligns with their mechanistic roles in inflammation and metabolism. While calibration deviations in IVDD may reflect cohort heterogeneity, the models’ clinical net benefit supports their utility as preliminary tools. Future studies with larger, prospectively collected datasets will refine these models.

Malic enzymes play a crucial role in the tricarboxylic acid (TAC) cycle by catalyzing the reversible conversion of malate to pyruvate (Jiang et al., 2013). These enzymes play crucial roles in NADPH production and redox homeostasis (Jiang et al., 2013). Previous have reported that malate was critical to decrease the secretion of pro-inflammatory cytokines and promote M2 phenotype, that inhibiting its production will activate inflammation (Zhang et al., 2023; Chen et al., 2024). Notably, in LPS-induced macrophages, ME1 is overexpressed, and its transcriptional regulation is governed by NF-κB (Santarsiero et al., 2024). Luo et al. (2024) also found that inactivating ME1 could effectively alleviate pulmonary hypertension. Our *in vitro* experiments also revealed that the administration of malate significantly ameliorated the generation of pro-inflammatory macrophages (Supplementary Figure S5A). Notably, treatment with inhibitor, ME1, showed similar anti-inflammatory effects (Supplementary Figure S5B). ME1 also play a critical role in regulating reactive oxygen species (ROS) level and cell viability (Fang et al., 2023; Brashears et al., 2022; Subbiah and Gan, 2022). Fang et al. (2023) discovered that ME1 functioned as a novel ferroptosis regulator by modulating NADPH homeostasis. However, the role of ME1 in IVDD has not been documented. Our findings revealed a significant upregulation of ME1 expression in both IVDD and NAFLD pathologies. Comprehensive analysis of bulk RNA sequencing data through KEGG pathway enrichment demonstrated distinct regulatory roles of ME1 in these conditions: in IVDD, ME1 predominantly modulates pathways associated with cell proliferation, ECM catabolism, and inflammatory responses, while in NAFLD, it primarily participates in metabolic regulation. These differential pathway involvements suggest a context-dependent functionality of ME1 in tissue homeostasis. Sc-RNA seq further confirmed the wide regulatory effects of ME1 on liver and IVD homeostasis. Importantly, exercise, while ameliorating the progression of NAFLD combined with IVDD, simultaneously influenced the expression of the ME1 gene. Based on these mechanistic insights, we propose that ME1 may represent a promising therapeutic target for maintaining the physiological equilibrium of both intervertebral disk and hepatic tissues, potentially offering a novel approach for managing these interconnected pathological conditions.

Hyaluronan synthase 2 (HAS2) is a crucial enzyme embedded in the cell membrane responsible for synthesizing hyaluronan (HA) (Sun et al., 2022). In addition, HAS2 modulates the tissue microenvironment and homeostasis (Rauhala et al., 2018). HAS2 is highly responsive to cell growth signals, such as glucocorticoid (Saavalainen et al., 2005), prostaglandins (Sussmann et al., 2004), and cytokines (Tammi et al., 2011). As a membrane-bound protein, HAS2 has an extracellular segment responsible for hyaluronan synthesis and an intracellular segment associated with cytoskeleton protein functions, which are crucial for cell survival and movement processes (Vigetti and Passi, 2014). HA synthesis by HAS2 has been shown to be crucial for the normal development of intervertebral disk, and the absence of this synthase lead to potential complications in the structural integrity of the spine (Roughley et al., 2011). In addition, HA has been widely used to promote tissue regeneration via hyaluronan-based hydrogels (Shen et al., 2022; Zhao et al., 2024; Liu et al., 2024). The impact of HAS2 on NAFLD has not been documented. However, HAS2 could promote carbon tetrachloride-induced acute and chronic inflammation in the liver (Kim et al., 2022). HAS2 could also mediate Notch1 activation and liver fibrosis (Yang et al., 2019). Our results indicated that HAS2 was primarily expressed in NPCs within IVD tissue, but with relatively limited expression in liver cell clusters. Notably, we found that HAS2 may serve as a shared diagnostic biomarker for the coexistence of IVDD and NAFLD, and can also be affected by exercise. However, considering the diverse roles of HAS2 in IVD and liver, further studies are needed to explore the biological mechanisms of HAS2.

The beta-2 adrenergic receptor (ADRB2) belongs to superfamily A of the seven-transmembrane G protein-coupled receptors (GPCRs) activated by the sympathetic nervous system (SNS) (Philipp and Hein, 2004). The formation of a β-arrestin-ADRB2 complex, induced by ligand binding, functions as a scaffold to activate multiple signaling pathways, such as MEK/ERK signaling (Lefkowitz, 2007). Peripheral sympathetic nerve fibers and their neurotransmitters have gained importance during degeneration over the past few decades (Grässel, 2014). A study on IVDD has reported that the localization of ADRB2 corresponds with ECM alterations, and that the expression of ADRB2 in IVD tissue decreases with the severity of IVDD (Kupka et al., 2020). Consistent with previous study, we also revealed a decreased expression of ADRB2 with increased severity of IVDD (Grässel, 2014). For NAFLD, ADRB2 gene was found to correlate closely with NAFLD (Loomba et al., 2010). Kang et al. (2023) also found that suppressing ADRB2 signaling promoted obesity and NAFLD. Our findings, along with previous studies, suggest that ADRB2 plays a significant role in IVDD and NAFLD. Furthermore, we have discovered that ADRB2 was also expressed in immune cells, including macrophages and T cells. In reality, sympathetic neurotransmitters exert a direct influence on immune cell dynamics through their corresponding receptors. According to Hasegawa et al. (2021), activation of sympathetic signaling in macrophages inhibits systemic inflammation and protects against renal ischemia–reperfusion injury. Recent studies have revealed that ADRB2 functioned as a novel checkpoint receptor, suppressing T cell-mediated anti-tumor responses. Moreover, a lack of ADRB2 has been shown to enhance CAR-T cell proliferation, increase the CD8/CD4 T cell ratio, and decrease apoptosis in these cells (Ajmal et al., 2024). KEGG analysis of bulk RNA sequencing of this study also demonstrated the regulatory effect of ADRB2 on inflammatory response. Notably, a recent study has demonstrated that aerobic exercise can activate ADRB2 signaling, thereby mitigating the progression of Alzheimer’s disease (AD) (Wu et al., 2024). Surprisingly, only a 30-min exercise could result in a significant upregulation of ADRB2 (Cho et al., 2015). Our *in vivo* experiment confirmed that exercise could significantly affect the expression of Adrb2 in both liver and IVD tissue. Thus, we proposed that engaging in adequate exercise can aid in addressing IVDD and NAFLD through ADRB2 signaling.

Three hub genes associated with inflammation and immunity were identified in the preliminary enrichment analysis. In patients with IVDD and NAFLD, ssGSEA revealed distinct patterns of immune cell infiltration. The IVDD samples displayed infiltration of 9 immune cell types, in contrast to the NAFLD samples which exhibited 18 types, underscoring significant differences when compared to the control group. Under normal circumstances, the intervertebral disk (IVD) tissue is considered an immune-privileged organ. However, extensive evidence has demonstrated the central role of macrophages during the development and progression of IVDD (Koroth et al., 2023). Macrophage polarization toward the M1 phenotype to initiate inflammation is the critical step to exacerbate IVDD, accompanied by death and senescence of NPCs (Koroth et al., 2023). Therefore, shift M1 (pro-inflammation) macrophages to M2 (anti-inflammation) macrophages become a promising strategy to delay IVDD (Koroth et al., 2023). Similarly, in NAFLD, liver macrophages have been shown to promote NAFLD in obesity through producing inflammatory mediators and inhibiting macrophage function could decrease inflammatory cytokine secretion and ameliorate NAFLD (Zhang et al., 2019; Barreby et al., 2022). However, unlike IVDD, the heterogeneity of macrophages in NAFLD encompasses their distinct ontogeny, ranging from embryonic Kupffer cells to bone marrow-or monocyte-derived macrophages, as well as their functional phenotypes (Barreby et al., 2022). Despite extensive research, no disease-modifying therapy has been established yet. Immunotherapy using targeting antibodies to regulate disease-associated macrophages holds promise, but a thorough understanding of these macrophage subtypes is essential for developing effective therapeutic approaches for IVDD and NAFLD.

This study pioneeringly identified three hub genes associated with IVDD and NAFLD, paving the way for further exploration of their molecular mechanisms. However, it is important to acknowledge several limitations here. Firstly, we identified only three hub genes, which may not provide a comprehensive understanding of the connection between IVDD and NAFLD. Additionally, the public datasets used were limited, and studies with larger clinical patient samples are needed. Secondly, considering the inherent differences in gene expression profiles across tissues, we did not directly compare gene expression profiles between the two tissue types. Instead, our comorbidity analysis was conducted through the following rigorous approach: (1) independent within-tissue analyses: for NAFLD, we compared liver gene expression between NAFLD patients vs. healthy controls; For IVDD, we compared intervertebral disk gene expression between IVDD patients vs. healthy controls, which will ensure all differential expression analyses were tissue-specific and biologically meaningful. (2) Identification of shared genes: In our study, only genes showing consistent expression difference (e.g., upregulated in both NAFLD liver and IVDD disk tissues) were considered as comorbidity candidates. This approach inherently accounts for tissue-specific baselines by focusing on disease-induced changes relative to each tissue’s own normal state. (3) Validation of functional relevance: further KEGG analyses confirmed the shared genes participate in conserved biological processes, such as inflammation across tissues. In addition, single-cell RNA-seq demonstrated that these hub genes maintain functional importance in disease-relevant cell types, such as ME1 in regulating macrophage function in both tissues. Thirdly, we acknowledge the deviations in calibration curves, particularly for IVDD. We deduced there are two possible reasons. Firstly, the IVDD cohorts (GSE153761, GSE56081, and GSE70362) are smaller and more heterogeneous than the NAFLD datasets, which may impact the model stability. Secondly, IVDD and NAFLD progression involves complex, multifactorial processes, such as mechanical stress, metabolism, or aging, beyond the scope of the three hub genes, which may limit predictive precision. In our further study, datasets with more samples will be required to enhance the efficacy of calibration curve for IVDD and NAFLD. Thirdly, although our work provides the first evidence linking Me1/Has2/Adrb2 to NAFLD-IVDD comorbidity, the specific molecular mechanisms through which these hub genes impact the diseases remain unclear. While we validated key comorbidity genes at the mRNA level, protein-level assays and functional experiments *in vitro*, such as gene editing or pharmacological modulation, were beyond the scope of this study. These will be prioritized in future work to establish causality. A robust bioinformatics-to-animal pipeline that can guide future research. Finally, while related studies have reported correlations between the hub genes and either IVDD or NAFLD, *in vivo* validation is also required to better reveal the critical mechanism of NAFLD involving in IVDD (Fang et al., 2023; Brashears et al., 2022; Yang et al., 2019; Kupka et al., 2020).

## Conclusion

Our results underscored ME1, HAS2, and ADRB2 as key diagnostic biomarkers for IVDD and NAFLD. These insights not only enhance our understanding of the intricate mechanisms at play in these diseases but also provide valuable direction for future research endeavors and clinical applications. Importantly, we preliminarily proved that alleviating NAFLD via swimming showed protective effects on IVDD in the context of HFD. The finding of our study highlights the close relationship between IVDD and NAFLD, and indicates that exercise, such as swimming, recommended to improve the NAFLD, as well as IVDD.

## Data Availability

The original contributions presented in the study are included in the article/Supplementary material, further inquiries can be directed to the corresponding authors.

## Footnotes

^1^ https://www.ncbi.nlm.nih.gov/

^2^ https://www.genecards.org/

^3^ http://bio-bigdata.hrbmu.edu.cn/CellMarker/
