# Supplementary material for: Cross-disease biomarker identification reveals shared diagnostic biomarkers for IVDD and NAFLD via bulk and single-cell RNA sequencing
Source: Front Mol Neurosci. 2025 Sep 22;18:1639705. doi: 10.3389/fnmol.2025.1639705 (PMC12497830; doi:10.3389/fnmol.2025.1639705)
Supplement: Supplementary file 1 [file Table_1.DOCX]

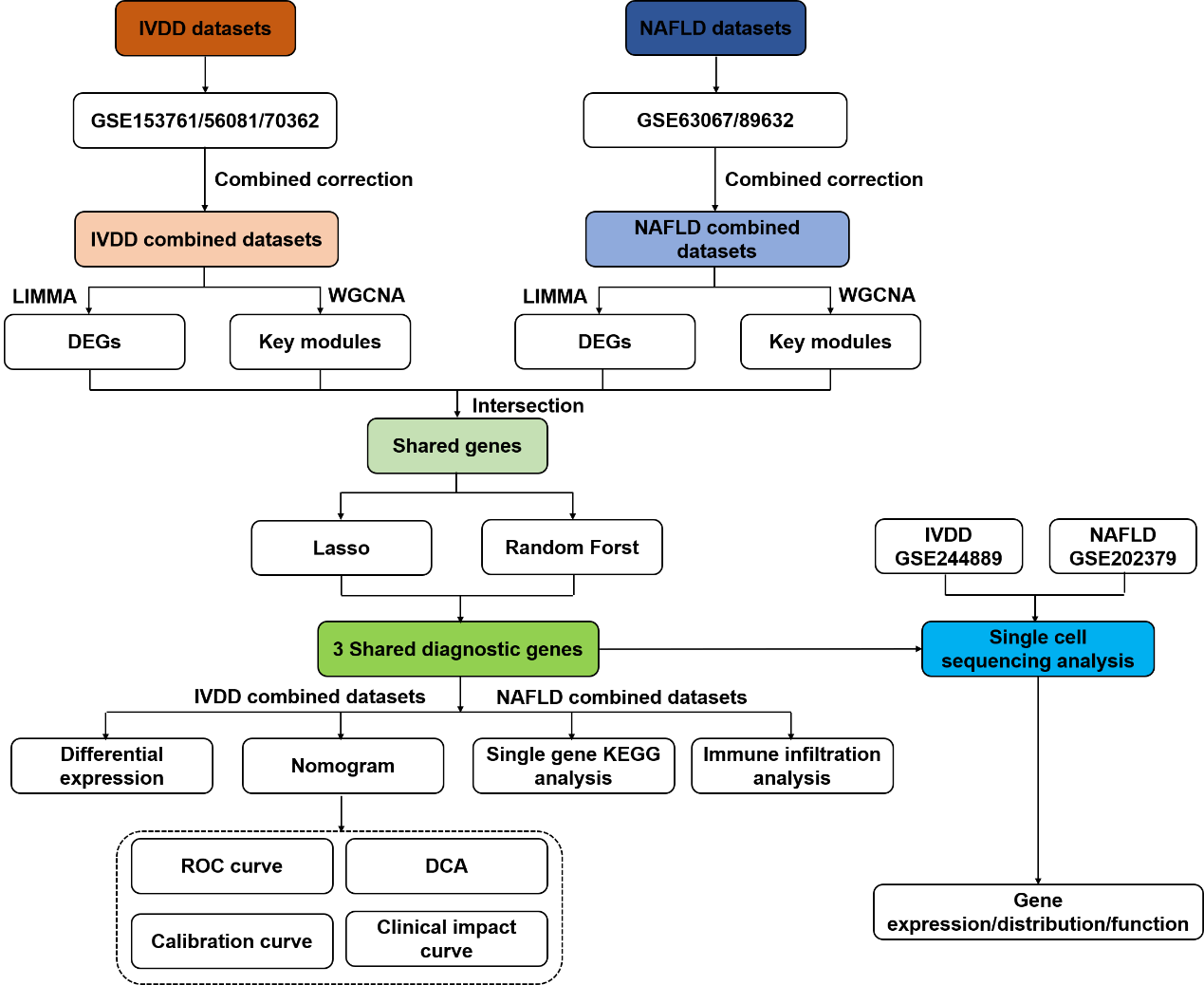


**Figure S1 Flowchart illustrating the study design.**


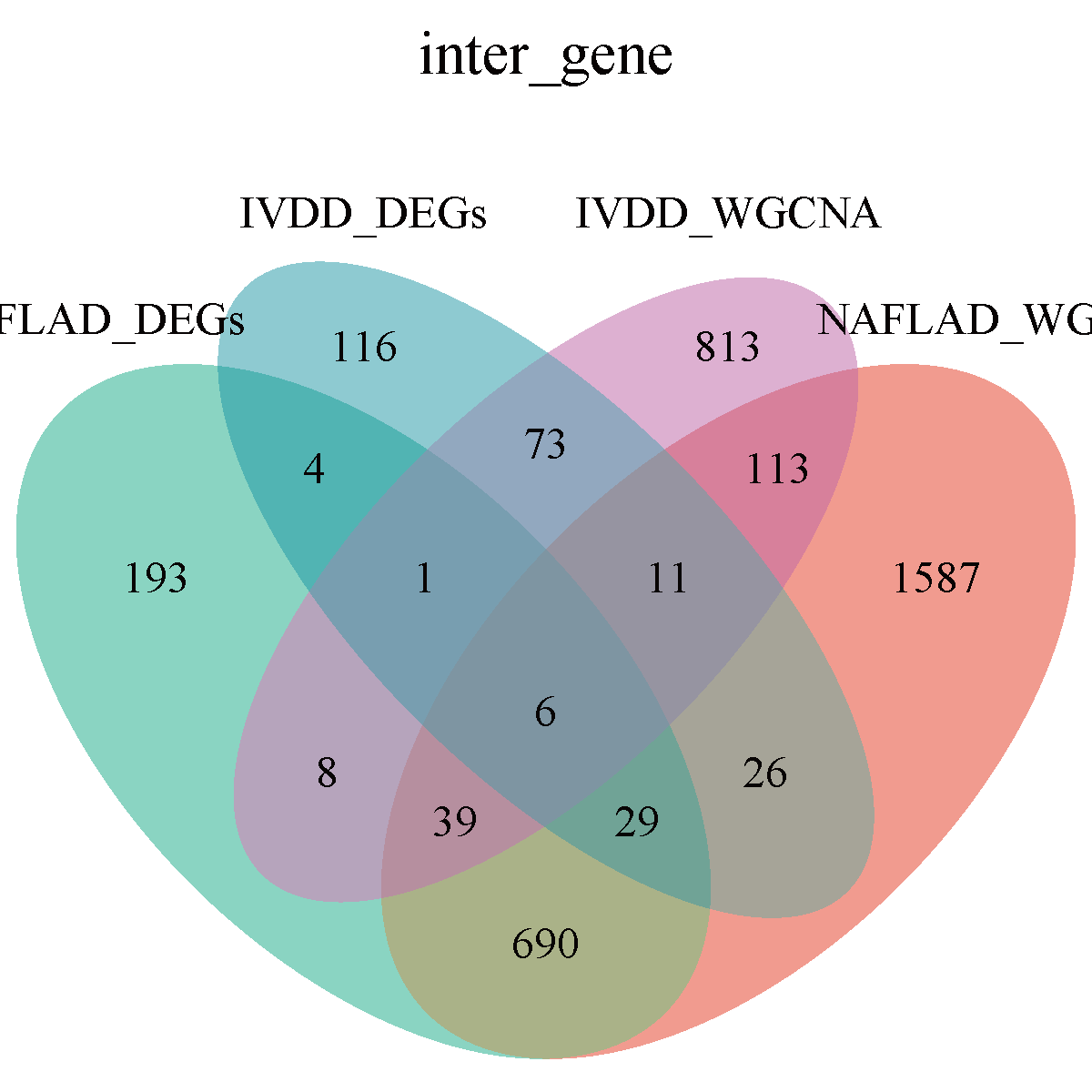


**Figure S2 Shared genes between IVDD and NAFLD identified by overlapping their DEGs and WGCNA.**


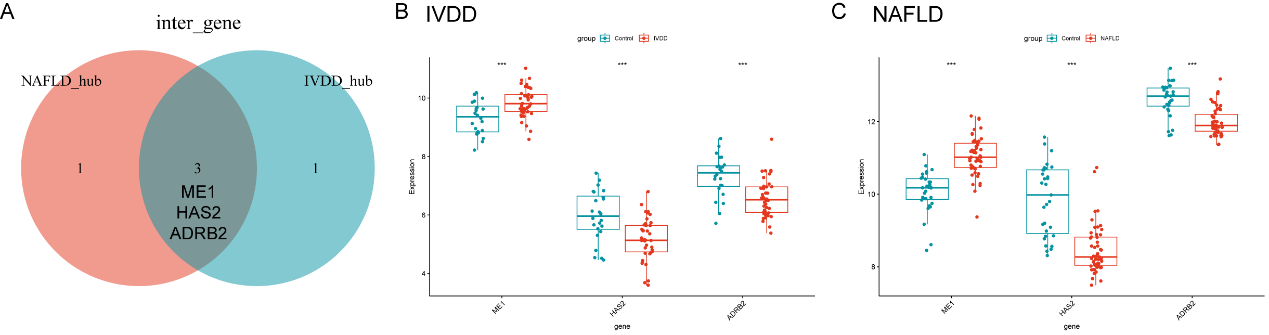


**Figure S3** A: Venn diagram revealed the two diagnostic genes in common between IVDD and NAFLD. B: Differential expression analysis of IVDD. C: Differential expression analysis of NAFLD.


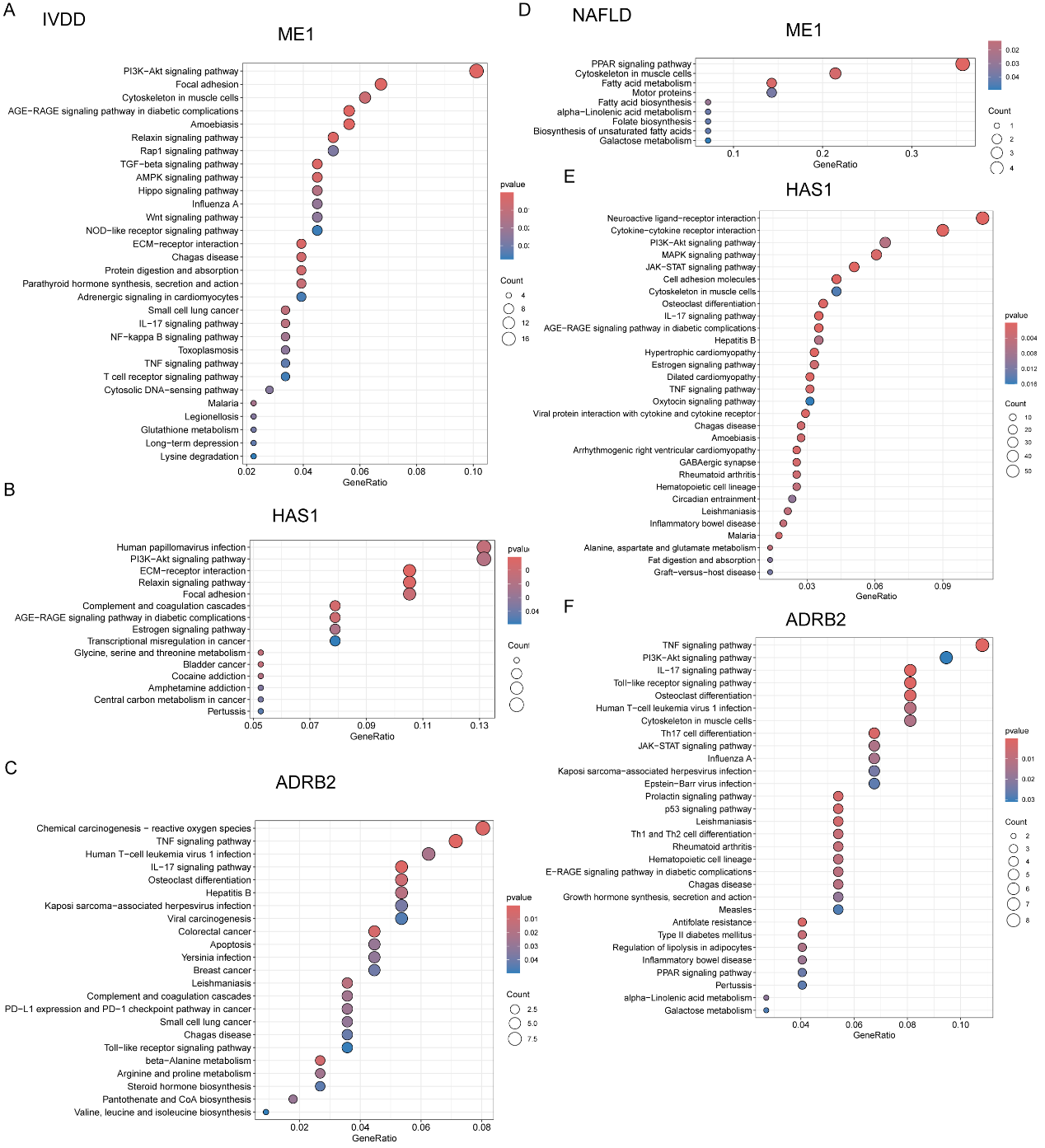


**Figure S4 KEGG for the single diagnostic gene**. A: KEGG analysis for ME1, HAS2, and ADRB2 in IVDD. B: KEGG analysis for ME1, HAS2, and ADRB2 in NAFLD.

IVDD, Intervertebral disc degeneration; NAFLD, non-alcoholic fatty liver disease; KEGG: Kyoto Encyclopedia of Genes and Genomes.


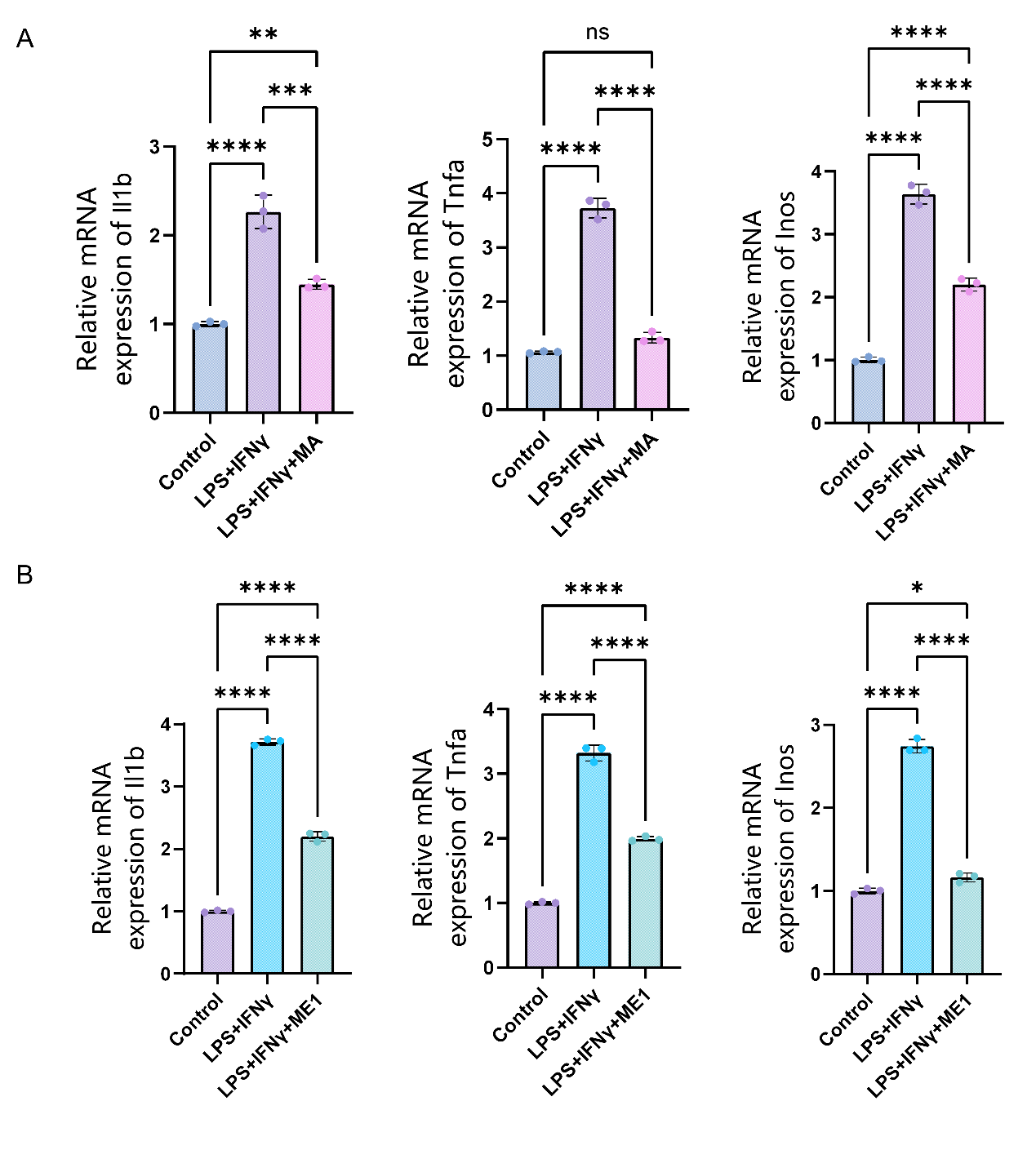


**Figure S5 The effects of Malate (MA) and ME1 inhibitor (ME1 or compound 1) on inflammatory macrophages** A: The effects of Malate (MA, 2 mM) on inflammatory macrophages; B: The effects of ME1 inhibitor (ME1 or compound 1, 10 μM) on inflammatory macrophages.
